# Analyzing the Impact of Gate Oxide Screening on Interface Trap Density in SiC Power MOSFETs Using a Novel Temperature-Triggered Method

**DOI:** 10.3390/mi16040371

**Published:** 2025-03-25

**Authors:** Monikuntala Bhattacharya, Michael Jin, Hengyu Yu, Shiva Houshmand, Jiashu Qian, Marvin H. White, Atsushi Shimbori, Anant K. Agarwal

**Affiliations:** 1 Department of Electrical & Computer Engineering, The Ohio State University, Columbus, OH 43210, USA; jin.845@osu.edu (M.J.); yu.3868@osu.edu (H.Y.); houshmand.3@osu.edu (S.H.); qian.539@osu.edu (J.Q.); white.1829@osu.edu (M.H.W.); agarwal.334@osu.edu (A.K.A.); 2 Ford Motor Co., Dearborn, MI 48124, USA; ashimbor@ford.com

**Keywords:** temperature-triggered threshold voltage shift method, interface trap distribution, gate oxide screening, SiC MOSFETs, planar vs. trench, cryogenic and high temperature

## Abstract

This work introduces a novel temperature-triggered threshold voltage shift (T3VS) method to study the energy-dependent $D_{it}$ distribution close to the conduction band edge in commercial 1.2 kV 4H-SiC MOSFETs with planar and trench gate structures. Traditional $D_{it}$ extraction methodologies are complicated and require sophisticated instrumentation, complex analysis, and/or prior information related to the device design and fabrication, which is generally unavailable to the consumers of commercial devices. This methodology merely utilizes the transfer characteristics of the device and is straightforward to implement. The $D_{it}$ analysis using the T3VS method shows that trench devices have significantly lower $D_{it}$ in comparison to the planar devices, making them more reliable and efficient in practical applications. Furthermore, this study examines the impact of a novel room temperature gate oxide screening methodology called screening with adjustment pulse (SWAP) on the $D_{it}$ distribution in commercial planar MOSFETs, utilizing the proposed T3VS method. The result demonstrates that the SWAP technique is aggressive in nature and can introduce new defect states close to the conduction band edge. Hence, additional care is needed during screening optimization to ensure the reliability and usability of the screened devices in the consequent applications.

## 1. Introduction

Silicon carbide metal oxide semiconductor field-effect transistors (SiC MOSFETs) are gaining attention for a diverse range of applications in energy, automotive, and industrial sectors due to their excellent material properties such as wide bandgap, high thermal conductivity, and superior breakdown characteristics. SiC power MOSFETs with planar gate structures were commercialized in 2011 [1] and continued to dominate the market. However, devices with asymmetric and double trench [1,2] gate designs are gaining prominence due to their enhanced channel mobility [1] and superior short circuit withstand time [2]. In reality, trench MOSFETs exhibit considerable reliability concerns attributable to their design and manufacturing process. At the trench corner, electric field crowding happens under high gate bias, leading to the degradation of the gate oxide reliability [3]. Additionally, the gate oxide in trench MOSFETs is deposited instead of being thermally grown like in planar MOSFETs, affecting the quality of the oxide [2]. The gate oxide processing technique affects the variability in the interface state density ($D_{it}$) at the SiC/SiO_2_ interface. The $D_{it}$ in SiC MOSFETs is almost two orders of magnitude higher compared to their Si counterparts [4], which poses both performance and reliability concerns. First, the inversion channel mobility of SiC devices is still in the range of 30–50 cm^2^/V-s [5,6,7], almost one order lower compared to the Si devices (>200 cm^2^/V-s). Additionally, the high $D_{it}$ leads to threshold voltage instability [8,9,10], jeopardizing the reliability of the device during operation.

On the other hand, the high density of extrinsic defects such as pits and holes, along with contamination in the oxide [11], poses significant issues in gate oxide reliability. The existence of these defects leads to high localized electric fields and/or increased current density within the gate oxide, as discussed in the oxide thinning model, leading to premature failure in the field [11,12]. As a result, proper screening needs to be implemented to effectively eliminate devices with poor gate oxide quality. Shi et al. [3] have demonstrated a high-voltage and high-temperature-based screening technique that can remove defective devices with negligible degradation in static characteristics such as threshold voltage ($V_{th}$) and intrinsic lifetime of the non-defective devices. But this methodology is limited to a screening oxide field ($E_{screen}$) of <9 MV/cm for a screening time ($t_{screen}$) of 100 ms–1 s to prevent a significant negative shift in $V_{th}$ due to charge trapping [3,13,14], which reduces the screening efficiency. Jin et al. [15] have proposed a novel room temperature screening method called screening with adjustment pulse (SWAP) that employs an $E_{screen}$ ≥ 9 MV/cm for a $t_{screen}$ of 10 s followed by an adjustment pulse ($E_{adj}$) of 8 MV/cm. This SWAP approach facilitates recovering the negative $V_{th}$ shift due to the high $E_{screen}$ by releasing the trapped holes while applying the $E_{adj}$ pulse. This method allows for more aggressive screening with higher screening efficiency without the need for high temperature.

One of the towering concerns related to the SWAP technique is the potential introduction of the additional interface states close to the conduction band edge as a consequence of the high gate oxide fields. As the presence of the interface states affects both the reliability and performance of SiC MOSFETs [16], multiple studies have been carried out to extract the energy-dependent $D_{it}$ profile, especially close to the conduction band edge, by employing different techniques such as thermal dielectric relaxation current (TDRC) and isothermal dielectric relaxation current (IDRC) [7,17,18], capacitance and conductance [6,19,20], thermally simulated current [21,22], subthreshold slope [23], and subthreshold hysteresis [24,25], or an amalgamation of two or more techniques. The standard capacitance-based $D_{it}$ extraction method, commonly referred to as the high–low method [6,26], is the most dominant among many methodologies. Nevertheless, it lacks the sensitivity to identify fast interface states. The sensitivity of $D_{it}$ extraction techniques can be enhanced by employing high-frequency input, low temperature, or equipment with extremely low-noise floors, or a combination of all of the above. Nevertheless, these procedures frequently exhibit limited adaptability and generally necessitate extremely sensitive apparatuses and/or intricate measurement and extraction processes [6].

In this paper we have proposed a novel and straightforward $D_{it}$ extraction technique, called the temperature-triggered threshold voltage shift (T3VS) method, which has been further verified using commercial 1.2 kV 4H-SiC power MOSFETs with planar and trench gate structures. In this technique, a $D_{it}$ distribution very close to the conduction band edge can be extracted by monitoring the variation in gate voltage with temperature at a constant drain current of 1 µA in a broad temperature range of 30 K to 450 K. Furthermore, this method is viable without needing the detailed knowledge of the device design or fabrication parameters. By utilizing the novel $D_{it}$ extraction methodology, this paper then examines the impact of the SWAP approach on the interface state density near the conduction band edge in commercial 1.2 kV planar SiC power MOSFETs.

## 2. Mathematical Analysis of the Temperature-Triggered Threshold Voltage Shift (T3VS) Method

**Figure 1:**
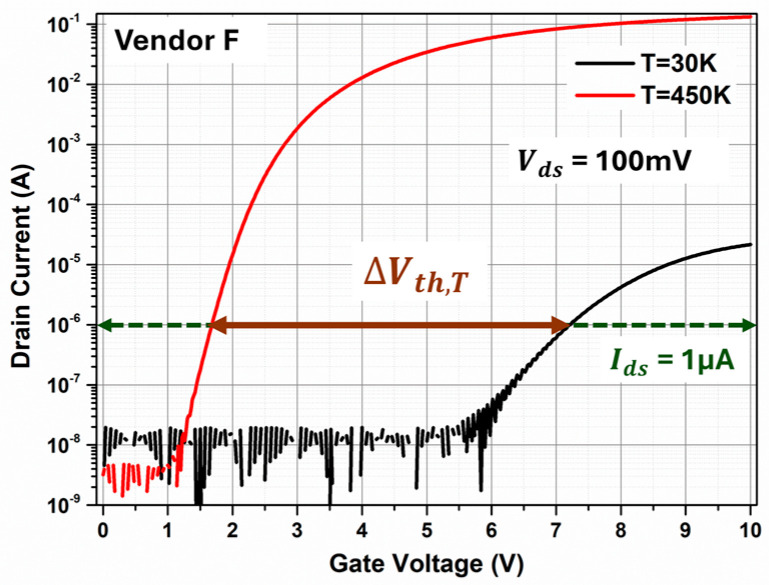
Transfer characteristics of Vendor F at T = 30 K and T = 450 K.

In order to evaluate the energy-dependent $D_{it}$ at the SiC/SiO_2_ interface by utilizing the T3VS method, first the effect of temperature on threshold voltage ($V_{th})$ needs to be examined. Figure 1 shows the transfer characteristics of a planar MOSFET from Vendor F at T = 30 K (black curve) and T = 450 K (red curve) under a constant drain voltage ($V_{ds}$) of 100 mV.

$V_{th}$ can be extracted by measuring the gate voltage ($V_{gs}$) at a constant drain current ($I_{ds}$) of 1 µA. Mathematically, $V_{th}$ can be written as [27],

(1) $$V_{th}={2\varphi }_{F}+\varphi_{SS}+\frac{\sqrt{4q\epsilon_{s}\varphi_{F}N_{A}}}{C_{ox}}-\frac{Q_{it}}{C_{ox}}-\frac{Q_{F}}{C_{ox}}$$

where $\varphi_{F}$ is the Fermi potential, $\varphi_{SS}$ is the Si-SiC work function difference, q is the elementary charge, $N_{A}$ is the acceptor concentration, $\epsilon_{s}$ is the permittivity of silicon carbide, $C_{ox}$ is the oxide capacitance per unit area, $Q_{F}$ is the fixed oxide charge per unit area, and $Q_{it}$ is the interface trap charge per unit area.

As the temperature reduces, the Fermi level ($E_{F}$) moves closer to the valence band ($E_{v}$) in the p-bulk, increasing the $\varphi_{F}$ [27]. The change in $\varphi_{F}$ and bandgap $\left(E_{g}\right)$ with temperature cumulatively affects the first three terms of (1) and has been found to be ∼0.2 V [18] for different commercial SiC power MOSFETs.

By neglecting the effect of temperature change on $Q_{F}$, the change in $V_{th}$ due to temperature (${∆V}_{th,T})$ can be described as

(2) $${∆V}_{th,T}\;∼\;\frac{{∆Q}_{it}}{C_{ox}}=\frac{{q∆N}_{it}}{C_{ox}}$$

where ${∆N}_{it}$ represents the change in the number of interface traps per unit area and can be expressed as

(3) $${∆N}_{it}=\int_{\varphi_{F,HT}}^{\varphi_{F,LT}}D_{it}(\varphi_{F})d\varphi_{F}$$

Combining (2) and (3) we obtain

(4) $$∆N_{it}=\int_{\varphi_{F,HT}}^{\varphi_{F,LT}}D_{it}(\varphi_{F})d\varphi_{F}=\frac{{∆V}_{th,T}C_{ox}}{q}$$

The energy distribution of the traps can be calculated as

(5) $${\left(E\right.}_{cs}-E_{T})=\frac{E_{g}}{2}-q\left(\varphi_{s}-\varphi_{F}\right)$$

where ${\left(E\right.}_{cs}-E_{T})$ is the position of the trap level relative to the conduction band edge at the surface and $\varphi_{s}$ is the surface potential within the range $\varphi_{F}<\varphi_{s}<{2\varphi }_{F}$. The distribution of $D_{it}$ (eV^−1^cm^−2^) with the variation in $E_{cs}-E_{T}$ (eV) can be extracted using (4) and (5).

## 3. Experimental Methodologies

### 3.1. Device Information

In this work, commercial 1.2 kV 4H-SiC MOSFETs with planar and trench gate structures were tested for $D_{it}$ extraction. In order to study the effect of SWAP on $D_{it}$, planar MOSFETs from Vendor F were considered. Prior to screening and $D_{it}$ experiments, a high-temperature (150 °C) gate oxide ramp-to-breakdown measurement was performed on a small sample from each vendor to estimate the gate oxide thickness ($t_{ox}$) considering the critical oxide electric field ($E_{crit}$) of 11 MV/cm [23,28,29]. The detailed information on the DUTs is listed in Table 1.

### 3.2. Experimental Methodology to Determine $D_{it}$

To determine $D_{it}$ distribution close to the conduction band edge using the T3VS method, DUTs were subjected to a large temperature variation of 30 K to 450 K. The low-temperature measurements (30 K–300 K) were carried out using a Lakeshore CCS-400/202 Cryostat (Lake Shore Cryotronics, Inc., Westerville, OH, USA). The DUT was maintained at a vacuum level of 0.1 mTorr while cooling inside the cryostat. High-temperature measurements (300 K–450 K) were conducted in a DX302 Yamato natural convection oven (Yamato Scientific Co. Ltd., Tokyo, Japan). Transfer characteristics were measured by using a Keysight B1506A Power Device Analyzer (Keysight Technologies, Colorado Springs, CO, USA). All measurements were performed once the temperature settled within 0.1 K of the target value.

### 3.3. SWAP Methodology

**Figure 2:**
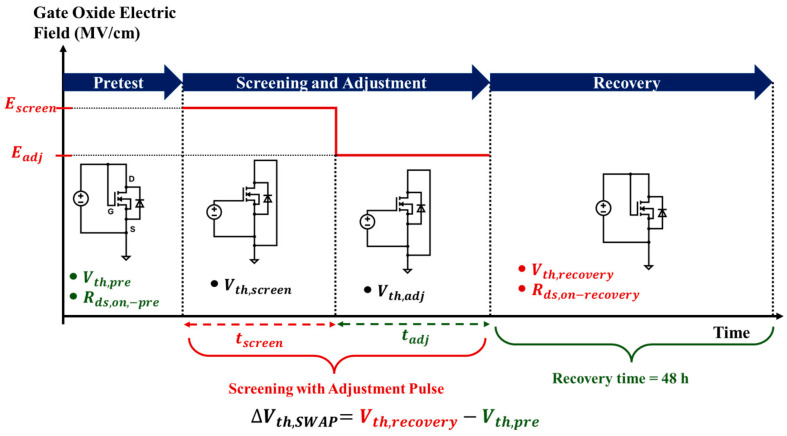
Test procedure of the SWAP methodology.

The gate oxide screening using SWAP methodology [15] for Vendor F was carried out at room temperature by utilizing a Keysight B1506A Power Device Analyzer (Keysight Technologies, Colorado Springs, CO, USA). The measurement methodology is graphically represented in Figure 2. At each step, the $V_{th}$ of the DUT was measured at a constant drain current of $I_{ds}$ = 1 mA. As a first step, each DUT was tested to determine $V_{th,pre}$. Then a screening pulse ($E_{screen}$) was applied for a time $t_{screen}$, followed by an adjustment pulse ${(E}_{adj})$ for time $t_{adj}$. The voltage corresponding to the electric field (for example, $V_{screen}$ related to $E_{screen}$, or $V_{adj}$ related to $E_{adj}$) was calculated by multiplying the electric field with the $t_{ox}$ of the device.

The measured threshold voltage after $E_{screen}$ is called $V_{th,screen}$ and after $E_{adj}\;$is termed as $V_{th,adj}.$ As a final step, the DUT was left to recover for 48 h at room temperature, and the threshold voltage after recovery ($V_{th,recovery}$) was then measured. The threshold voltage shift due to the SWAP screening (${∆V}_{th,SWAP}$) and percentage of threshold voltage shift (${\%V}_{th,SWAP})$ can be defined as

(6) $${∆V}_{th,SWAP}=V_{th,recovery}-V_{th,pre}$$

(7) %Vth,SWAP=∆Vth,SWAPVth,pre×100%.

The effect of SWAP screening on the on-resistance ($R_{ds,on})$ of each DUT was monitored by measuring $R_{ds,on}$ at $V_{gs}$ = 20 V and $V_{ds}$ = 1.5 V. The on-resistance shift due to screening (${∆R}_{ds,on-SWAP}$) and ${\%∆R}_{ds,on-SWAP}$ is defined as

(8) $${∆R}_{ds,on-SWAP}=R_{ds,on-recovery}-R_{ds,on-pre}$$

where $R_{ds,on-pre}$ is the pretest on-resistance and $R_{ds,on-recovery}$ is the on-resistance of the device after screening and 48 h of recovery.

(9) %∆Rds,on−SWAP=∆Rds,on−SWAPRds,on−pre×100%

## 4. Results and Discussion

### 4.1. $D_{it}$ Extraction of Commercial 1.2 kV 4H-SiC MOSFETs Using T3VS Technique

This section shows the energy-dependent $D_{it}$ distribution of commercial 1.2 kV 4H-SiC MOSFETs with planar and trench gate structures close to the conduction band edge, extracted using the T3VS method. Since this methodology is based on the $V_{th}$ shift as a function of temperature as shown in (2), the baseline $V_{th}$ and corresponding baseline $D_{it}$ profile as a function of ${\left(E\right.}_{cs}-E_{T})$ need to be established at a baseline temperature ($T_{baseline}$). In this analysis, we have considered $T_{baseline}$ = 450 K. The subsequent investigation has been discussed below.

#### 4.1.1. Effect of Temperature on $V_{th}$ of the DUTs

**Figure 3:**
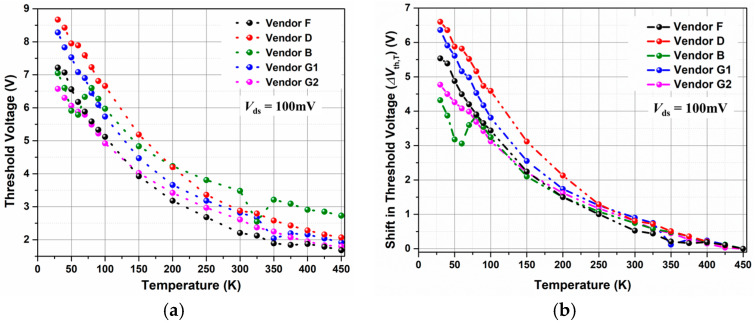
Variation in (**a**) threshold voltage and (**b**) ${∆V}_{th,T}$ as a function of temperature for all vendors.

Figure 3a shows the variation in $V_{th}$ extracted at $I_{ds}$ = 1 µA with respect to temperature (T) for all the DUTs. By considering the $V_{th}$ at $T_{baseline}$ = 450 K as the baseline value, the shift in threshold voltage due to temperature (${∆V}_{th,T}$) can be defined as

(10) $${∆V}_{th,T}=V_{th,T}-V_{th,450K}$$

where $V_{th,T}$ is the threshold voltage measured at a given temperature in K.

**Figure 4:**
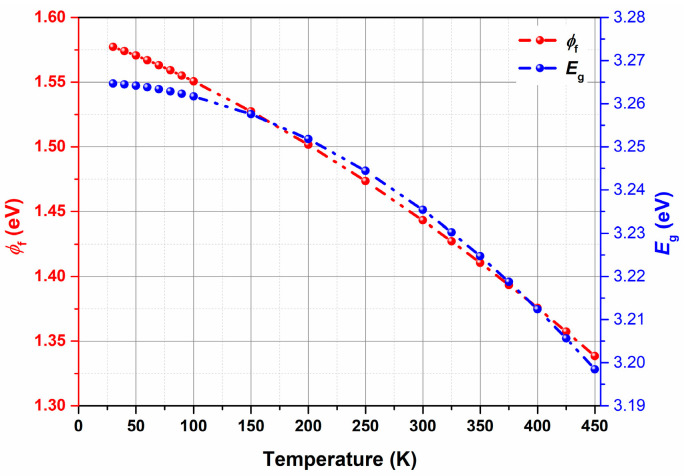
Variation in $\varphi_{F}$ and *E_g_* with temperature considering $N_{A}$ = 2 × 10^17^/cm^3^.

The variation in ${∆V}_{th,T}$ for all the DUTs as a function of temperature is shown in Figure 3b. This large shift in threshold voltage (for example, ${∆V}_{th,30K}$ = 5.534 V for Vendor F) cannot be accounted for only by the temperature dependency of $\varphi_{F}$ and *E_g_*. Utilizing theoretical understanding [30] and considering $N_{A}$ = 2 × 10^17^/cm^3^ [27], the variation in $\varphi_{F}$ and *E_g_* as a function of temperature has been calculated and shown in Figure 4. It can be clearly seen that the maximum shift in $\varphi_{F}$ and *E_g_* is ∼0.25 eV and 0.07 eV, respectively, when the temperature shifts from 450 K to 30 K.

#### 4.1.2. Baseline $D_{it}$ Profile Extraction

As a next step, the baseline $D_{it}$ profile was evaluated at $T_{baseline}$ = 450 K by using the subthreshold region of the transfer characteristics [23] of the DUT at 450 K and is termed as $D_{it,450K}$. The methodology is discussed below.

The subthreshold region drain current

(11) $$I_{DS}=I_{0}e^{\frac{qV_{gs}}{nkT}}(1-e^{\frac{qV_{ds}}{kT}})$$

and the ideality factor *n* is given as

(12) $$n=\frac{q}{2.3kT}{\left(\frac{\partial logI_{DS}}{\partial V_{gs}}\right)}^{-1}=1+\frac{C_{D}+C_{it}}{C_{ox}}$$

where $C_{D}$ is the depletion capacitance, $C_{ox}$ is the oxide capacitance, and $C_{it}$ is the interface trap capacitance per unit area.

The energy-dependent interface trap density is given as

(13) $$D_{it}=\frac{C_{it}}{q}$$

and the energy distribution of the trap level ${(E}_{cs}-E_{T})$ is

(14) $$E_{cs}-E_{T}=\frac{E_{g}}{2}-q(\varphi_{F}-\frac{2.3kT}{q}\log\left[\frac{I_{DS}\left(2\varphi_{F}\right)}{I_{DS}\left(\varphi_{s}\right)}\right])$$

where the surface potential ($\varphi_{s}$) is in the range of $\varphi_{F}\;$< $\varphi_{s}$ < ${2\varphi }_{F}$, and $\varphi_{F}$ is the Fermi potential.

**Figure 5:**
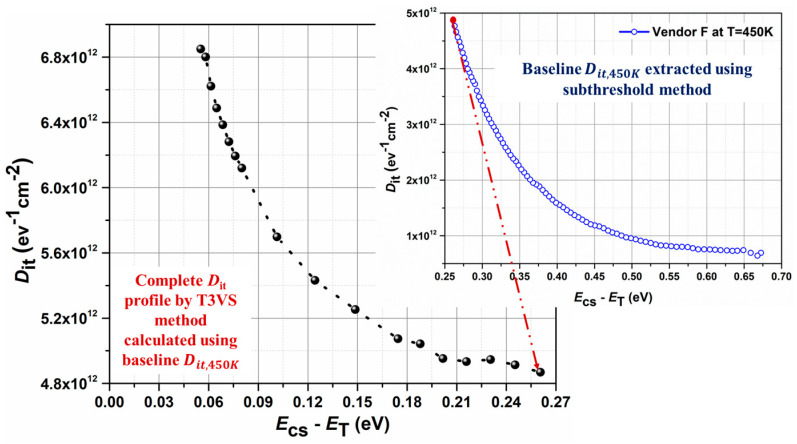
$D_{it}$ profile as a function of ${\left(E\right.}_{cs}-E_{T})$ extracted using the proposed T3VS method for Vendor F. The baseline $D_{it}$ profile extracted at $T_{baseline}$ = 450 K using the subthreshold method [23] is shown in the inset. The red line correlates the $D_{it,450K}$ with the complete $D_{it}$ profile.

Using (13) and (14), $D_{it,450K}$ has been extracted for Vendor F and shown in Figure 5 (inset). Using the baseline $D_{it,450K}$ along with (4) and (5), the energy-dependent $D_{it}$ distribution profile very close to the conduction band edge (${\left(E\right.}_{cs}-E_{T})$ ∼0.05 eV) has been determined for Vendor F and is presented in Figure 5.

**Figure 6:**
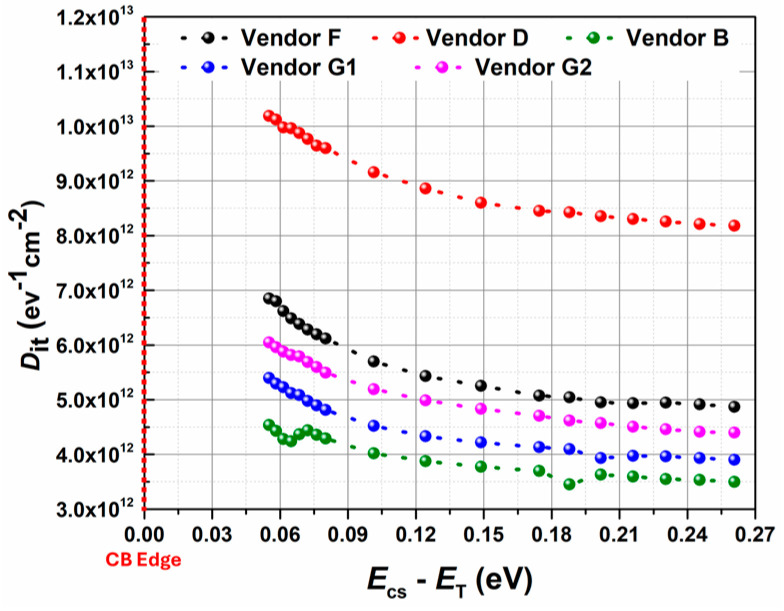
The extracted $D_{it}$ profile as a function of ${\left(E\right.}_{cs}-E_{T})$ for all the vendors.

The extracted $D_{it}$ profile, as shown in Figure 5, agrees with the previously reported $D_{it}$ profiles close to the conduction band edge in SiC/SiO_2_ systems [19,31], demonstrating this technique is both appropriate and reliable for use in commercial 1.2 kV SiC MOSFETs. Therefore, similar analysis was carried out for all the vendors, and the $D_{it}$ distribution profile as a function of ${\left(E\right.}_{cs}-E_{T})$ has been extracted and shown in Figure 6. It can be seen that devices with planar gate structures (red and black curves) show higher $D_{it}$ compared to devices with trench gate structures (pink, blue, and green curves). As a result, trench devices exhibit higher carrier density in the channel region [1]. Furthermore, the lower $D_{it}$ very close to the conduction band edge (${\left(E\right.}_{cs}-E_{T})$ ∼0.05 eV) for trench devices results in improved channel mobility and threshold voltage stability, making them more attractive for real-world applications. Although this method is suitable to extract trap distribution very close to the conduction band edge, it fails to provide any information related to the trap properties, trap parameters such as thermal activation energy, capture cross section, or the origin of the trap states.

### 4.2. Effect of SWAP on $D_{it}$ Distribution

To study the effect of screening on interface state density close to the conduction band edge in commercial devices using the T3VS method, a small batch of devices from Vendor F were subjected to the SWAP screening method followed by the $D_{it}$ analysis. The $D_{it}$ profile of the screened devices was compared with an unscreened one from the same batch with the same part and lot number, and the results are discussed below.

**Figure 7:**
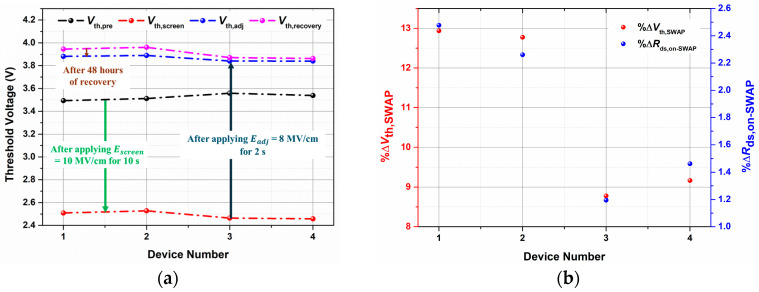
(**a**) The variation in threshold voltage during the SWAP process; (**b**) The percentage shift in threshold voltage and on-resistance due to the SWAP process.

For the SWAP screening, we have applied the highest possible $E_{screen}$ = 10 MV/cm for $t_{screen}$ = 10 s, followed by an $E_{adj}$ = 8 MV/cm for $t_{adj}$ = 2 s [15], and the variation in $V_{th}$ at each step has been measured and displayed in Figure 7a. The variation in ${\%∆V}_{th,SWAP}$ and ${\%∆R}_{ds,on-SWAP}$ has been calculated using (7) and (9), respectively, and shown in Figure 7b. It is evident that the maximum shifts in ${\%∆V}_{th,SWAP}$ and ${\%∆R}_{ds,on-SWAP}$ are 12.9% and 2.47%, respectively, for D1. Although the shift in on-resistance is within the permissible limit of ±5% [15] of their initial value, the shift in threshold voltage is significant.

**Figure 8:**
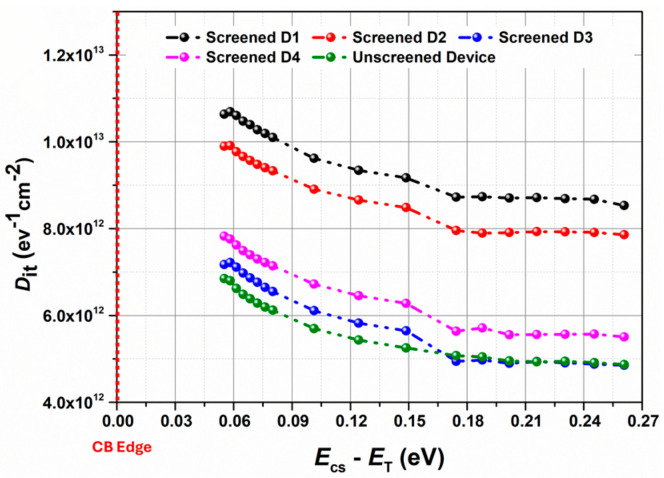
Extracted $D_{it}$ profile as a function of ${\left(E\right.}_{cs}-E_{T})$ for screened (black, red, pink, and blue curve) and unscreened (green curve) devices from Vendor F.

This substantial shift in threshold voltage can be attributed to either a significant number of electrons injected and subsequently trapped in the gate oxide or the creation of new interface defect states [32] during the SWAP screening process. The increase of threshold voltage has also raised the on-resistance as shown in Figure 7b. The effect of SWAP screening on the interface states has been further studied by comparing the $D_{it}$ profiles of the screened devices with respect to the $D_{it}$ profile of an unscreened device from the same vendor with the same part and lot number using the novel T3VS method as shown in Figure 8.

It can be clearly seen that device D1, with the highest ${\%∆V}_{th,SWAP}$ and ${\%∆R}_{ds,on-SWAP}$, results in maximum degradation of the interface by introducing new defect states close to the conduction band edge, i.e., 0.05 eV ≤ ${\left(E\right.}_{cs}-E_{T})$≤ 0.26 eV. In order to better understand the degradation of the interface state due to SWAP screening, the change in the number of traps (${\Delta N}_{it}$) has been further calculated and is presented below.

**Figure 9:**
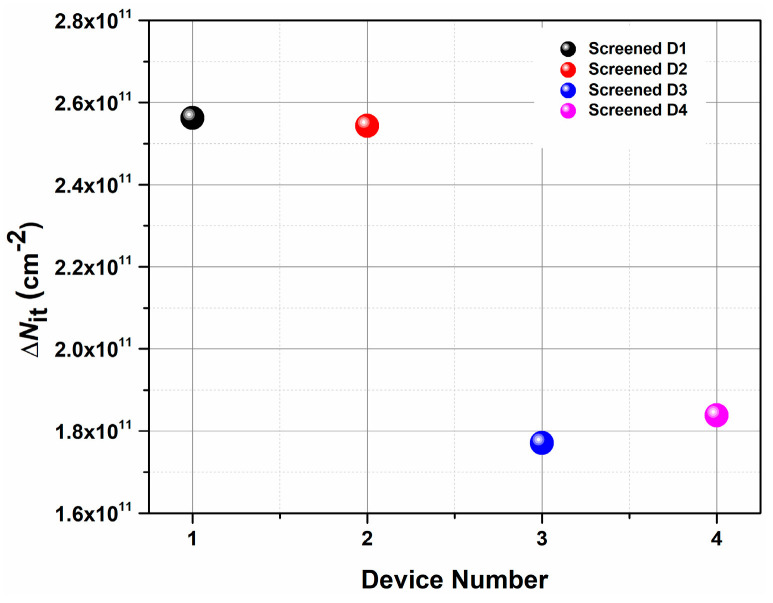
Extracted ${\Delta N}_{it}$ for screened devices from Vendor F.

Figure 9 shows the variation in the extracted ${\Delta N}_{it}$ for all the screened devices from Vendor F. It can be clearly seen that the increase in the total number of traps is significant (∼10^11/^cm^2^) under all the screening conditions. Therefore, it is possible to assert that the substantial alteration in threshold voltage is mostly attributed to the emergence of new defect states near the conduction band edge, which further prevents the restoration of the devices to their pristine state and hence affects their usability in ensuing applications. Moreover, it can be inferred that to implement the SWAP screening method on an industrial scale, the screening conditions need to be calibrated extremely carefully to prevent the formation of new defect states close to the conduction band edge so that the screened devices can be subsequently implemented in real-world applications.

## 5. Conclusions

This paper introduces a novel and simple temperature-triggered threshold voltage shift (T3VS) method to study the interface state density close to the conduction band edge in 1.2 kV 4H-SiC power MOSFETs with planar and trench gate structures. This approach solely uses the transfer characteristics of the MOSFETs, making it incredibly straightforward to employ. Furthermore, no prior information related to the device design or fabrication process is needed to implement this technique. Then, this work studies the effect of a novel room temperature gate oxide screening methodology known as the SWAP technique in light of the interface defect state analysis. The $D_{it}$ analysis on unscreened and screened planar devices from Vendor F further reveals that the SWAP screening is extremely aggressive in nature and can create new defect states close to the conduction band edge, which will harm the usability of the screened devices. In order to implement SWAP screening in practical scenarios, extreme care needs to be taken during the screening optimization to prevent the generation of any new defect states that could impair the reliability of the devices in real-world applications.

## Figures and Tables

**Table 1:** Details of 1.2 kV SiC Power MOSFETs.

Vendor	Structure	$\mathrm{Estimated}\;t_{ox}$ [nm]	Current Rating [A]	On-Resistance [mΩ]
F	Planar	38	7.6	350
D	Planar	45	20	189
B	Asymmetric trench	57.1	4.7	350
G1	Double trench	58.1	17	160
G2	Reinforced double trench	39.6	26	62

## Data Availability

Data are contained within this article.

## Abbreviations

The following abbreviations are used in this manuscript:

T3VS	temperature-triggered threshold voltage shift
SWAP	screening with adjustment pulse
MOSFET	metal oxide semiconductor field-effect transistor
DUT	device under test
